# Supplementary material for: Quantum Vibronic Effects on the Electronic Properties of Molecular Crystals
Source: J Chem Theory Comput. 2023 Jun 28;19(13):4011–22. doi: 10.1021/acs.jctc.3c00424 (PMC10339675; doi:10.1021/acs.jctc.3c00424)
Supplement: Supplementary file 1 — ct3c00424_si_001.pdf [file ct3c00424_si_001.pdf]

SUPPORTING INFORMATION
Quantum vibronic effects on the electronic properties of molecular crystals

Arpan Kundu*,† and Giulia Galli*,‡,†,¶

†*Pritzker School of Molecular Engineering, The University of Chicago, Chicago, Illinois 60637, United States*

‡*Department of Chemistry, University of Chicago, Chicago, Illinois 60637, United States*

¶*Materials Science Division and Center for Molecular Engineering, Argonne National Laboratory, Lemont, Illinois 60439, United States*

E-mail: arpan.kundu@gmail.com; gagalli@uchicago.edu

Contents

S1 Methods **S2**

S1.1 Theory S2

S1.2 Stochastic Approach S6

S1.3 Frozen Phonon Approach S7

S2 Additional Figures **S10**

S2.1 Mode Resolved EPCEs and Anharmonic Measures S10

S2.2 Effect of torsion on the potential energy surface and the band gap of an isolated NAI-DMAC molecule S14

References S18

S1 Methods

S1.1 Theory

Within the Born-Oppenheimer (BO) approximation, the Schrödinger equation for the quantum nuclear motion of a system with N nuclei can be expressed as,[1]

$$\hat{\mathcal{H}}|\chi_k(\mathbf{R})\rangle = \left(-\frac{1}{2}\sum_I \frac{1}{M_I}\nabla^2_{R_I} + \mathcal{V}(\mathbf{R})\right)|\chi_k(\mathbf{R})\rangle = \varepsilon_k|\chi_k(\mathbf{R})\rangle, \tag{S1}$$

where M_I and $\mathbf{R} = (R_{1x}, R_{1y}, ..., R_{Nz})$ are the mass of the I-th nucleus and the Cartesian position vector of all nuclei with respect to a chosen origin, respectively. $\mathcal{V}(\mathbf{R})$, and $|\chi_k(\mathbf{R})\rangle$ represent the $3N$-dimensional adiabatic potential energy surface, and the wave function for the k-th nuclear state, respectively. Since, $\mathcal{V}(\mathbf{R})$ and $|\chi_k(\mathbf{R})\rangle$ are indifferent to the choice of the origin, for the convenience of discussion, we set the origin at the coordinates of the equilibrium geometry where $\mathcal{V}(\mathbf{R})$ is minimum. We consider only the Γ-point vibrations within the simulation cell.

We consider an electronic observable, e.g., electronic eigenenergies or total energy, $\mathcal{O}(\mathbf{R}) = \langle\psi(\mathbf{r};\mathbf{R})|\hat{\mathcal{O}}(\mathbf{r};\mathbf{R})|\psi(\mathbf{r};\mathbf{R})\rangle$, that depends on one electronic state. Here $\mathbf{r}$ and $|\psi(\mathbf{r};\mathbf{R})\rangle$ are the electronic coordinates and wave function, respectively. We note that as a consequence of BO approximation, the electronic wave function has only parametric dependence on the nuclear coordinates, $\mathbf{R}$. When the system is at equilibrium at temperature T, the effect of electron-phonon interaction on the electronic property $\mathcal{O}(\mathbf{R})$ can be included by performing an ensemble average over all adiabatic nuclear states k,

$$\langle\mathcal{O}\rangle_T = \frac{1}{Q(T)}\sum_{k=0}^{\infty}\langle\chi_k(\mathbf{R})|\hat{\mathcal{O}}(\mathbf{R})|\chi_k(\mathbf{R})\rangle \exp\left(-\frac{\varepsilon_k}{k_BT}\right) = \int d\mathbf{R}W(\mathbf{R},T)\mathcal{O}(R). \tag{S2}$$

Here,

$$W(\mathbf{R},T) = \frac{1}{Q(T)} \sum_{k=0}^{\infty} \langle \chi_k(\mathbf{R}) | \chi_k(\mathbf{R}) \rangle \exp\left(-\frac{\varepsilon_k}{k_B T}\right), \tag{S3}$$

denotes the probability of finding the system with the nuclear coordinates within $\mathbf{R}$ and $\mathbf{R} + d\mathbf{R}$. The partition function Q is defined as,

$$Q(T) = \sum_{k=0}^{\infty} \exp\left(-\frac{\varepsilon_k}{k_B T}\right), \tag{S4}$$

where k_B is the Boltzmann constant.

A molecular dynamics simulation with either a path-integral approach[2,3] or quantum thermostat approach[4–6] utilizes Eq. S2 to compute the electron-phonon renormalized electronic properties. However, being computationally expensive, such simulations are rarely adopted for computing electron-phonon renormalizations from first principles. The standard approaches in solid-state physics employ approximations to $\mathcal{V}(\mathbf{R})$, to simplify the expression in Eq. S2.

A Taylor series expansion of $\mathcal{V}(\mathbf{R})$ near the equilibrium geometry, $\mathbf{R} = \mathbf{0}$, and subsequently, neglecting the terms beyond second order yields the potential energy with harmonic approximation (HA),

$$\mathcal{V}^{\mathrm{H}}(\mathbf{R}) = \frac{1}{2} \sum_{I\alpha,J\alpha'} (R_{I\alpha}\sqrt{M_I}) D_{I\alpha,J\alpha'} (\sqrt{M_J} R_{J\alpha'}) = \frac{1}{2} \mathbf{R}^T \mathbf{M}^{1/2} \mathbf{D} \mathbf{M}^{1/2} \mathbf{R}, \tag{S5}$$

with $\mathbf{M}$ representing a $3N \times 3N$ diagonal matrix of nuclear masses. We note that the first order term of the Taylor expansion becomes zero by setting the condition of the minimum, $\frac{\partial \mathcal{V}(\mathbf{R})}{\partial R_{I\alpha}} = 0$. In addition, we set the reference potential energy, $\mathcal{V}(\mathbf{0}) = 0$. The elements of the dynamical matrix, which is also known as the mass-weighted Hessian matrix is given by,

$$D_{I\alpha,J\alpha'} = \frac{1}{\sqrt{M_I M_J}} \frac{\partial^2 \mathcal{V}(\mathbf{R})}{\partial R_{I\alpha} \partial R_{J\alpha'}}\bigg|_{\mathbf{R}=\mathbf{0}}, \tag{S6}$$

where α,α' denote the cartesian axes x,y or z and I, J denote the indices of the nuclei.

Spectral decomposition of the dynamical matrix, $\mathbf{D} = \mathbf{U}\mathbf{\Omega}^2\mathbf{U}^T$, returns a unitary matrix, $\mathbf{U}$, and a $3N \times 3N$ diagonal matrix of normal-mode frequencies, $\mathbf{\Omega}$, with diagonal elements: $\omega_1, \omega_2, ..., \omega_{3N}$. The unitary matrix, $\mathbf{U}$, defines the cartesian to normal mode transformations,

$$\begin{aligned} \mathbf{X} &= \mathbf{U}^T\mathbf{M}^{1/2}\mathbf{R}, \\ \nabla_X^2 &= \mathbf{U}^T\mathbf{M}^{1/2}\nabla_R^2, \end{aligned} \tag{S7}$$

and back transformation to cartesian from normal modes,

$$\begin{aligned} \mathbf{R} &= (\mathbf{M}^{1/2})^{-1}\mathbf{U}\mathbf{X}, \\ \nabla_R^2 &= (\mathbf{M}^{1/2})^{-1}\mathbf{U}\nabla_X^2, \end{aligned} \tag{S8}$$

with $\mathbf{X}$ representing the matrix of $3N$ normal mode vectors.

After transformation to normal modes, the total nuclear Hamiltonian separates into 3 independent translational degrees of freedom, d_r number of independent global rotational degrees of freedom, and $3N - 3 - d_r$ number of independent vibrational degrees of freedom. For a solid, linear isolated molecule, and a non-linear isolated molecule, the number of rotational degrees of freedom (d_r) is 0, 2, and 3, respectively. Since the translations and global rotations, which usually appear as the first $3 + d_r$ lowest eigenvalues, do not affect the electronic properties, we focus on the vibrational Hamiltonian which becomes,

$$\hat{\mathcal{H}}^{\mathrm{H}} = \sum_{\nu=3+d_r+1}^{3N} \left(-\frac{1}{2}\nabla_{X_\nu}^2 + \frac{1}{2}\omega_\nu^2 X_\nu^2 \right). \tag{S9}$$

The wavefunctions, $|\chi_{\nu,k}^{\mathrm{H}}\rangle$, and energies, $\varepsilon_{\nu,k}^{\mathrm{H}}$ of each simple harmonic oscillator is known analytically,

$$\langle X_\nu | \chi_{\nu,k}^{\mathrm{H}} \rangle = \frac{1}{\sqrt{2^k k!}} \left(\frac{\omega_\nu}{\pi} \right)^{\frac{1}{4}} \exp\left[\frac{-\omega_\nu X_\nu^2}{2} \right] H_k(\sqrt{\omega_\nu} X_\nu), \tag{S10}$$

$$\varepsilon_{\nu,k}^{\mathrm{H}} = \left(k + \frac{1}{2} \right) \omega_\nu, \tag{S11}$$

with H_k denoting the k-th order Hermite polynomial. Inserting the expression of $\varepsilon_{\nu,k}^{\mathrm{H}}$ from

Eq. S11 into Eq. S4 yields, the partition function for the ν-th harmonic oscillator,

$$Q_\nu^{\mathrm{H}}(T) = \sum_{k=0}^{\infty} \exp\left[-\frac{\omega_\nu}{k_B T}(k+\frac{1}{2})\right] = \exp\left(\frac{\omega_\nu}{2k_B T}\right) n_B(\omega_\nu, T), \tag{S12}$$

where the Bose occupation factor is given by,

$$n_B(\omega, T) = \frac{1}{\exp(\omega/k_B T) - 1} \tag{S13}$$

Because of the separation of Hamiltonian into $3N-3-d_r$ number of independent vibrational terms, the total vibrational wave function which is characterized by a vector of quantum numbers $\mathbf{k} = (k_{3+d_r+1}, k_{3+d_r+2}, ..., k_{3N})$ can be simplified by the product of the wave functions for each individual normal mode. The total partition function can be written analogously.

$$\begin{aligned} |\chi_{\mathbf{k}}^{\mathrm{H}}(R)\rangle &= \prod_{\nu=3+d_r+1}^{3N} |\chi_{\nu,k_\nu}^{\mathrm{H}}(R)\rangle, \\ Q^{\mathrm{H}}(T) &= \prod_{\nu=3+d_r+1}^{3N} Q_\nu^{\mathrm{H}}(T). \end{aligned} \tag{S14}$$

Utilizing the expressions from Eqs.S10–S14, Eq. S2 can be rewritten as,

$$\langle \mathcal{O} \rangle_T^{\mathrm{H}} = \int d\mathbf{X} W^{\mathrm{H}}(\mathbf{X}, T)\mathcal{O}(\mathbf{X}) = \int d\mathbf{X} \left[\prod_{\nu=3+d_r+1}^{3N} G(X_\nu; \sigma_{\nu,T})\right] \mathcal{O}(\mathbf{X}) \tag{S15}$$

where the harmonic probability density, $W^{\mathrm{H}}(\mathbf{X}, T)$, reduces to a product of independent Gaussian functions,

$$G(X_\nu; \sigma_{\nu,T}) = \frac{1}{\sqrt{2\pi\sigma_{\nu,T}^2}} \exp\left(-\frac{X_\nu^2}{2\sigma_{\nu,T}^2}\right), \tag{S16}$$

with widths related to the Bose occupation factor:

$$\sigma_{\nu,T} = \sqrt{\frac{2n_B(\omega_\nu, T) + 1}{2\omega_\nu}}. \tag{S17}$$

We note that though Eq. S15 is a result of harmonic approximation, it does not assume any explicit dependence of electronic observable $\mathcal{O}$ on nuclear coordinates ($\mathbf{R}$) or normal mode coordinates ($\mathbf{X}$). To further simplify the expression, we expand $\mathcal{O}(\mathbf{X})$ in Taylor series,

$$\mathcal{O}(\mathbf{X}) = \mathcal{O}(\mathbf{0}) + \sum_{\nu} \left.\frac{\partial \mathcal{O}}{\partial X_\nu}\right|_{\mathbf{0}} X_\nu + \sum_{\nu\nu'} \left.\frac{\partial^2 \mathcal{O}}{\partial X_\nu \partial X_{\nu'}}\right|_{\mathbf{0}} X_\nu X_{\nu'} + ... \tag{S18}$$

and truncate the expansion after the second order. After inserting the resulting expression into Eq.S15, we obtain the phonon renormalized electronic observable with quadratic (Q) approximation,

$$\langle \mathcal{O} \rangle_T^{\mathrm{Q}} = \mathcal{O}(\mathbf{0}) + \sum_{\nu=3+d_r+1}^{3N} \frac{1}{2\omega_\nu} \left.\frac{\partial^2 \mathcal{O}}{\partial X_\nu^2}\right|_0 \left[n_B(\omega_\nu, T) + \frac{1}{2} \right]. \tag{S19}$$

We note that terms involving odd order of X_ν or cross-coupling terms such as $X_\nu X_{\nu'}$, with $\nu \neq \nu'$, do not appear because harmonic density is symmetric with respect to $\mathbf{X} = \mathbf{0}$. For systems with strong anharmonicity, the vibrational density is no longer symmetric, and subsequently, both odd-order terms and cross-coupling terms become essential.

S1.2 Stochastic Approach

The stochastic approach employs Monte Carlo sampling of $W(\mathbf{X}, T)$ and subsequently utilizes Eq. S15 to compute phonon-renormalized electronic properties. In each Monte Carlo step, a displaced coordinate in normal mode is obtained, $\mathbf{X}^i = \boldsymbol{\tau}^i$, where, for $\nu > 3 + d_r$, the matrix elements, τ_ν^i, are a Gaussian distributed random number with zero mean and width $\sigma_{\nu,T}$, while the first $3 + d_r$ matrix elements are set to zero. Afterward, $\mathbf{X}^i$'s are back-transformed to Cartesian coordinates, $\mathbf{R}^i$, using Eq.S8, and subsequently, electronic observable $\mathcal{O}(\mathbf{R}^i)$ is computed. After M Monte Carlo steps, Eq. S15 can be re-written as,

$$\langle \mathcal{O} \rangle_T^{MC} = \frac{1}{M} \sum_{i=1}^{M} \mathcal{O}(\mathbf{X}^i) = \frac{1}{M} \sum_{i=1}^{M} \mathcal{O}(\mathbf{R}^i) \tag{S20}$$

Based on the Mean-value (MV) theorem and utilizing quadratic (Q) approximation, Monserrat proposed that there exists 2^{3N-3-d_r} mean-value positions, $\mathbf{X}_{\mathrm{MVQ}}$, for which $\mathcal{O}(\mathbf{X}_{\mathrm{MVQ}}) \simeq \langle\mathcal{O}\rangle_T$,[7] with $\mathbf{X}^i_{\mathrm{MVQ}} = \mathbf{s}^i\boldsymbol{\sigma}_T$, where the matrix elements of $\boldsymbol{\sigma}_T$ is given by Eq. S17, and $\mathbf{s}^i$ is a matrix with first $3 + d_r$ elements set to zero and remaining $3N - 3 - d_r$ elements are either +1 or -1. It was shown that a Monte Carlo algorithm that samples random signs, s^i, has a faster convergence of $\langle\mathcal{O}\rangle_T^{MC}$ than the one that samples random numbers, $\boldsymbol{\tau}^i$, from a Gaussian distribution.[7] Later, Zacharius and Giustino proposed a one-shot (OS) algorithm,[8] in which the signs are chosen according to,

$$\begin{aligned} s_\nu &= (-1)^{\nu-4-d_r} \text{ for } \nu > 3 + d_r \\ &= 0, \text{ for } \nu \leq 3 + d_r \end{aligned} \tag{S21}$$

and they showed that only a single first-principle calculation on the obtained atomic configuration, $\mathbf{X} = \mathbf{s}\boldsymbol{\sigma}_T$ is sufficient to converge of $\langle\mathcal{O}\rangle_T$. They also proposed that an additional first-principles calculation on the antithetic pair of the chosen atomic configuration, i.e., $\mathbf{X} = -\mathbf{s}\boldsymbol{\sigma}_T$, can improve the result, which we adopted here.

$$\langle\mathcal{O}\rangle_T^{OS} = \frac{1}{2}[\mathcal{O}(\mathbf{s}\boldsymbol{\sigma}_T) + \mathcal{O}(-\mathbf{s}\boldsymbol{\sigma}_T)] \tag{S22}$$

S1.3 Frozen Phonon Approach

A frozen phonon (FP) approach utilizes Eq. S19 to compute phonon-renormalized electronic properties. To compute the first and second derivatives of the observable, $\mathcal{O}'$, $\mathcal{O}''$, respectively, we displaced the nuclei to $X_\nu = +h_\nu$ and $X_\nu = -h_\nu$ along each phonon coordinates, and subsequently, used the central difference formula.

$$\mathcal{O}'(X_\nu = 0) = \left.\frac{\partial\mathcal{O}(X_\nu)}{\partial X_\nu}\right|_{X_\nu=0} = \frac{\mathcal{O}(+h_\nu) - \mathcal{O}(-h_\nu)}{2h_\nu} \tag{S23}$$

$$\mathcal{O}''(X_\nu = 0) = \left.\frac{\partial^2 \mathcal{O}(X_\nu)}{\partial X_\nu^2}\right|_{X_\nu=0} = \frac{\mathcal{O}(+h_\nu) + \mathcal{O}(-h_\nu) - 2\mathcal{O}(0)}{h_\nu^2}, \tag{S24}$$

We chose the displacements, h_ν, such that the potential energy difference, $\delta\mathcal{V}^H$, is the same for all modes, assuming a parabolic dependence of potential energy on each normal mode, i.e.,

$$h_\nu = \frac{\sqrt{2\delta\mathcal{V}^H}}{\omega_\nu}. \tag{S25}$$

Throughout this work, our electronic observable ($\mathcal{O}$) would be either valence and conduction band (VB) energies, E_n, with n denoting the band index, or the band gap, E_g. The second derivative of n-th band energies with respect to ν-th phonon mode scaled by the phonon frequency, which appears in the Eq. S19 when $\mathcal{O} = E_n$, is called the electron-phonon coupling energy (EPCE) of band n with respect to phonon mode ν.

$$\mathrm{EPCE}_{n,\nu} = \frac{1}{2\omega_\nu} \left.\frac{\partial^2 E_n}{\partial X_\nu^2}\right|_0 \tag{S26}$$

It is evident from Eq. S19, the electron-phonon renormalization of n-th band,

$$\Delta E_n(T) = \langle E_n \rangle_T - E_n(\mathbf{0}) \tag{S27}$$

reduces to,

$$\Delta E_n^Q(0) = \sum_{\nu=3+d_r+1}^{N} \mathrm{EPCE}_{n,\nu} \tag{S28}$$

within quadratic approximation at 0 K. EPCEs can be computed using the FP method and it describes the contributions of each mode towards the total electron-phonon renormalization of band energies.

Depending on the displacement chosen for each phonon mode, nearby eigenstates may cross each other, as we shall see during the discussion. In such cases, while calculating $\left.\frac{\partial E_n}{\partial X_\nu}\right|_0$ and $\left.\frac{\partial^2 E_n}{\partial X_\nu^2}\right|_0$ by applying Eq. S23 and S24, respectively, it is necessary to ensure that the electronic eigenvalues used for the positive and negative displacements correspond to

the same eigenstate. Following earlier works,[9,10] we ensured the correct correspondence by projecting wavefunctions with negative displacements on wavefunctions with positive displacements.

S2 Additional Figures

S2.1 Mode Resolved EPCEs and Anharmonic Measures

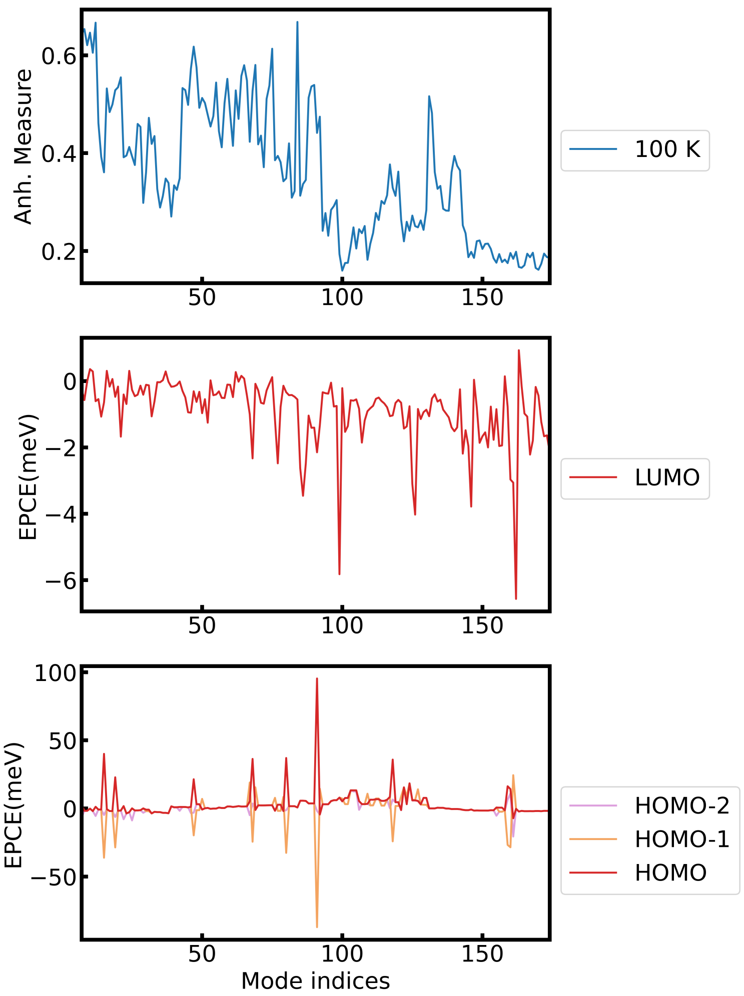

Figure S1: Mode-resolved anharmonic measures and electron-phonon coupling energies (EPCEs) of the lowest and highest occupied molecular orbitals for an isolated pentamantane molecule as obtained with the SCAN functional. **Top panel:** Mode-resolved anharmonic measures computed from the trajectory obtained with the quantum thermostatted molecular dynamics simulations at 100 K. **Middle panel:** Mode-resolved EPCEs of the lowest unoccupied molecular orbital (LUMO) computed using the frozen phonon approach. **Bottom panel:** Mode-resolved EPCEs of the highest occupied molecular orbital (HOMO), HOMO-1, and HOMO-2 levels computed using the frozen phonon approach.

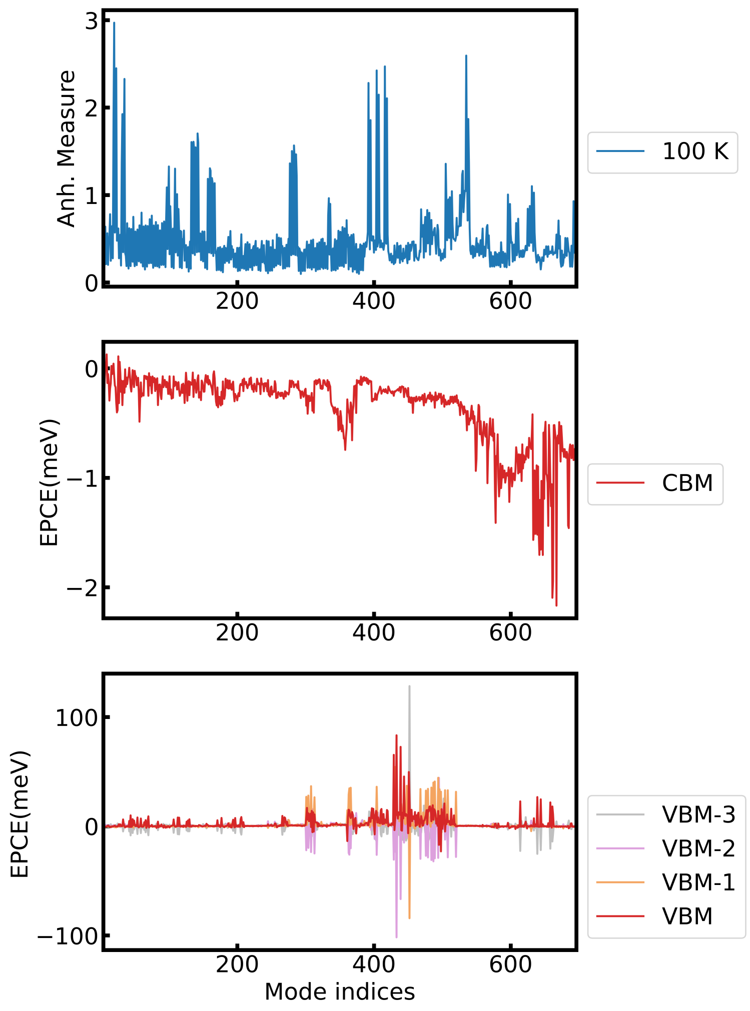

Figure S2: Mode-resolved anharmonic measures and electron-phonon coupling energies (EPCEs) of the valence and conduction bands for the pentamantane molecular crystal as obtained with the SCAN functional. **Top panel:** Mode-resolved anharmonic measures computed from the trajectory obtained with the quantum thermostatted molecular dynamics simulations at 100 K. **Middle panel:** Mode-resolved EPCEs of the conduction band minimum (CBM) computed using a frozen phonon calculation. **Bottom panel:** Mode-resolved EPCEs of the valence band maximum (VBM), VBM-1, VBM-2 and VBM-3 levels computed using the frozen phonon approach.

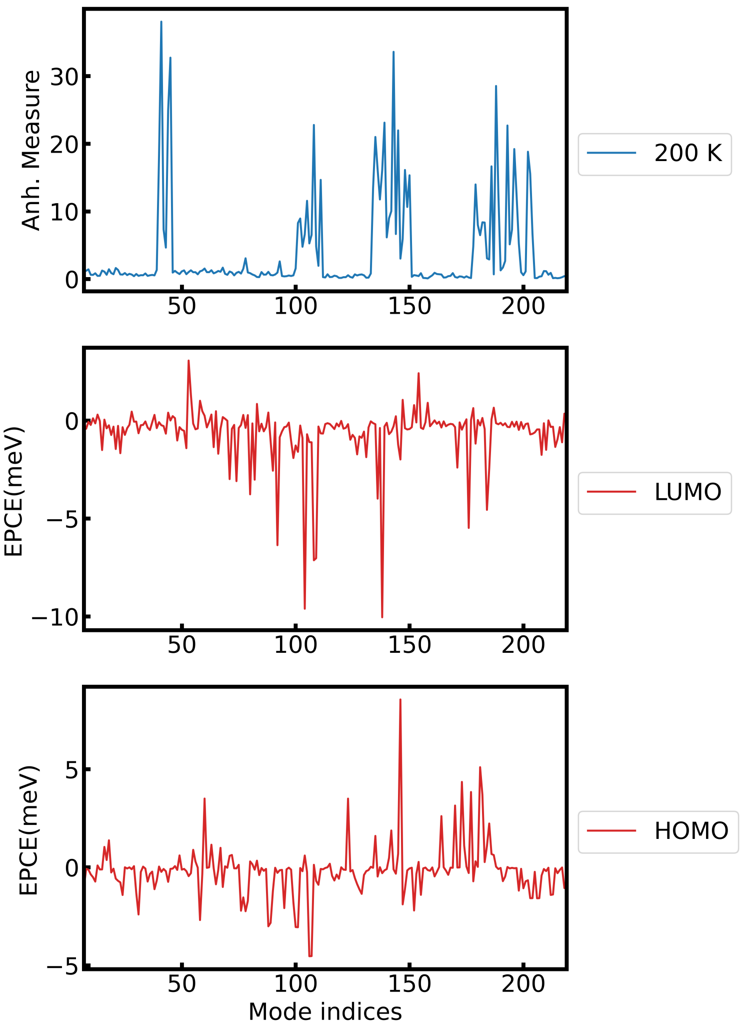

Figure S3: Mode-resolved anharmonic measures and electron-phonon coupling energies (EPCEs) of the lowest and highest occupied molecular orbitals for an isolated NAI-DMAC molecule as obtained with the PBE functional. **Top panel:** Mode-resolved anharmonic measures computed from the trajectory obtained with the quantum thermostatted molecular dynamics simulations at 200 K. **Middle panel:** Mode-resolved EPCEs of the lowest unoccupied molecular orbital (LUMO) computed using the frozen phonon approach. **Bottom panel:** Mode-resolved EPCEs of the highest occupied molecular orbital (HOMO) computed using the frozen phonon approach.

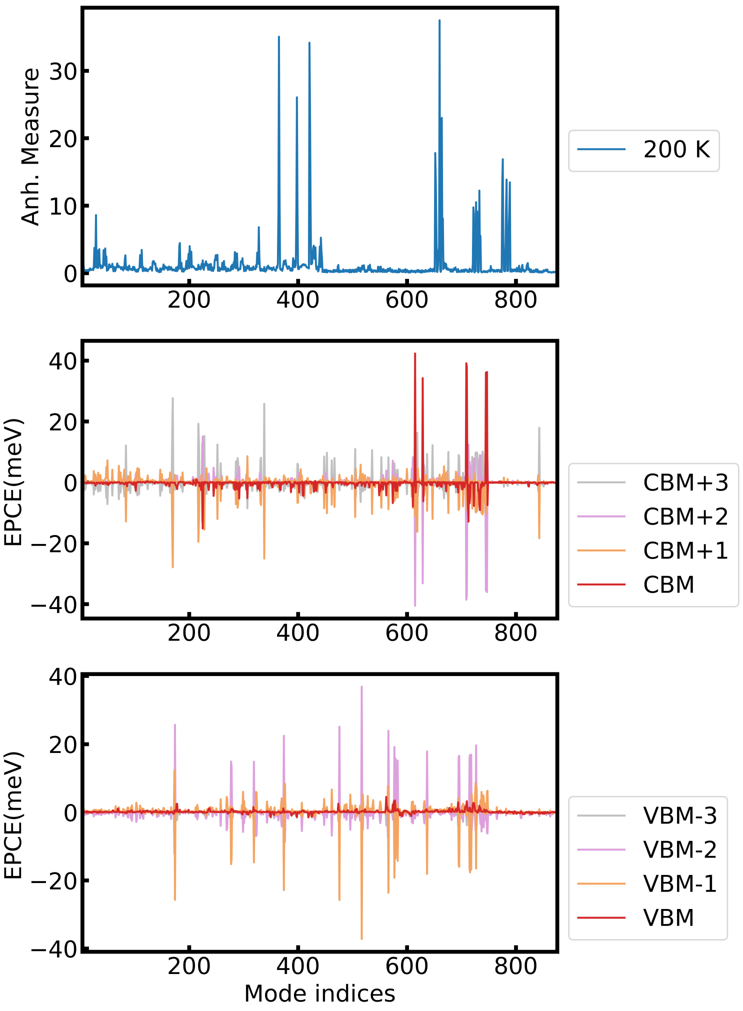

Figure S4: Mode-resolved anharmonic measures and electron-phonon coupling energies (EPCEs) of the valence and conduction bands for NAI-DMAC molecular crystal as obtained with the PBE functional. **Top panel:** Mode-resolved anharmonic measures computed from the trajectory obtained with the quantum thermostatted molecular dynamics simulations at 200 K. **Middle panel:** Mode-resolved EPCEs of the conduction band minimum (CBM), CBM+1, CBM+2, and CBM+3 levels computed using the frozen phonon approach. **Bottom panel:** Mode-resolved EPCEs of the valence band maximum (VBM), VBM-1, VBM-2 and VBM-3 levels computed using the frozen phonon approach.

S2.2 Effect of torsion on the potential energy surface and the band gap of an isolated NAI-DMAC molecule

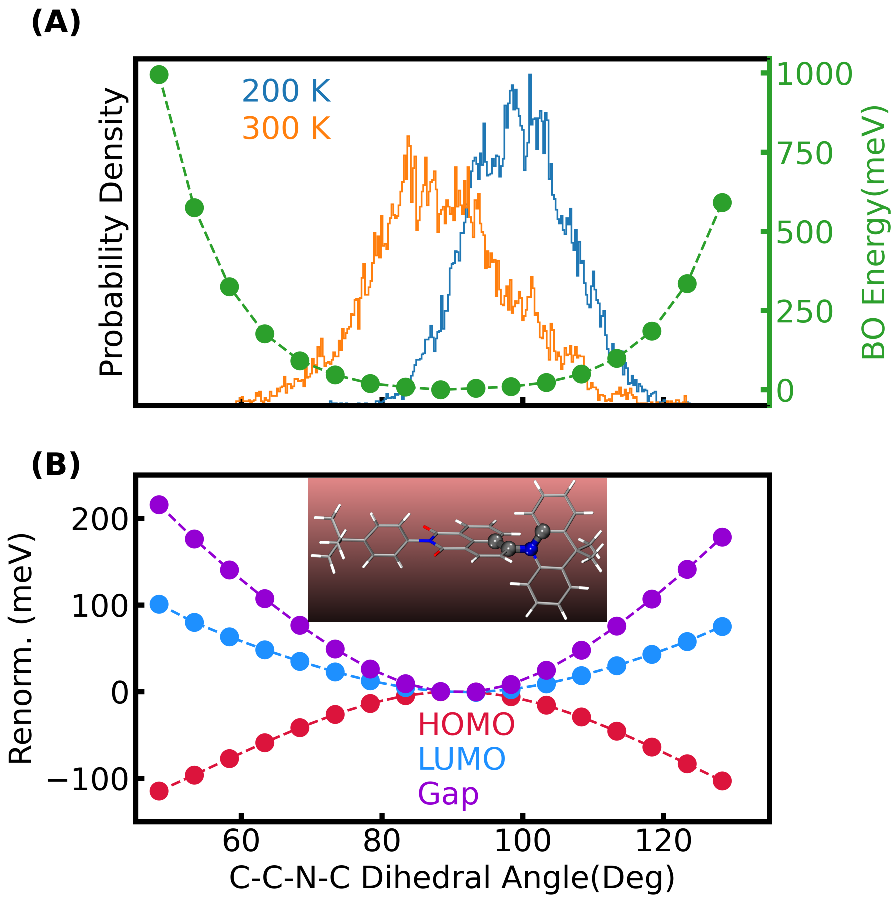

Figure S5: Panel A: Probability distribution of the C-C-N-C dihedral angle (highlighted as spheres in the inset of panel B) for the isolated molecule at different temperatures as obtained from QTMD simulations. The green circles represent the Born-Oppenheimer energy when PES is scanned by varying the dihedral angle for the isolated molecule. Panel B: The HOMO, LUMO, and fundamental renormalizations for the isolated molecule when only the dihedral angle is varied. See also Figs. 5,6 and 9 of ref.[11] and the discussions therein.

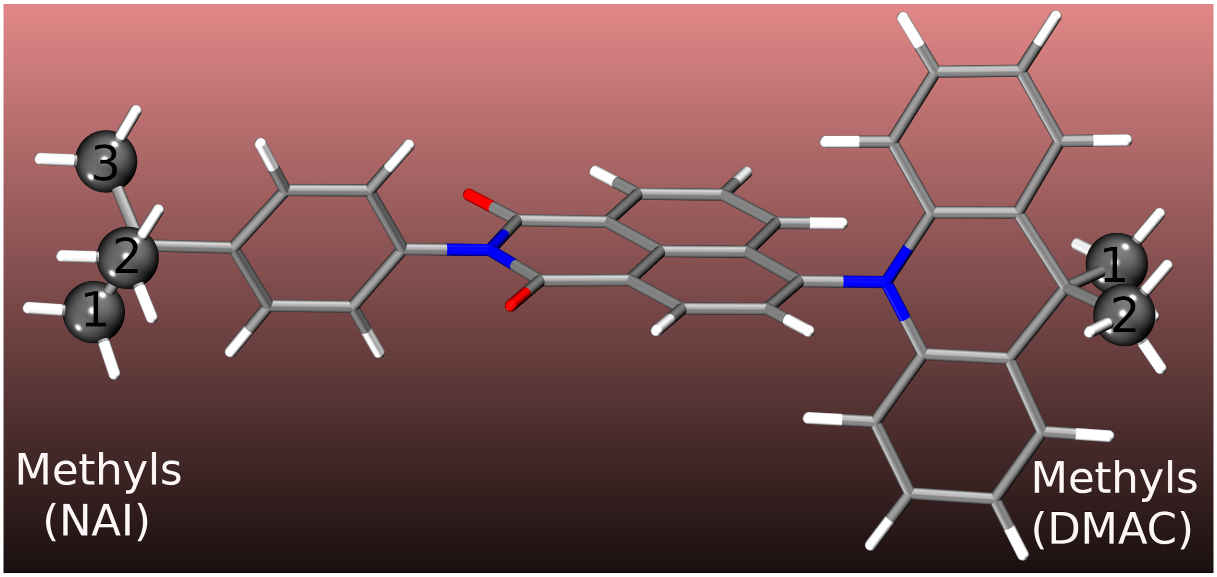

Figure S6: Three and two methyl groups of the NAI and DMAC units, respectively, of the isolated NAIDMAC molecules that are rotated relative to the geometry optimized positions. The central carbon atoms of these methyl groups are highlighted as gray spheres.

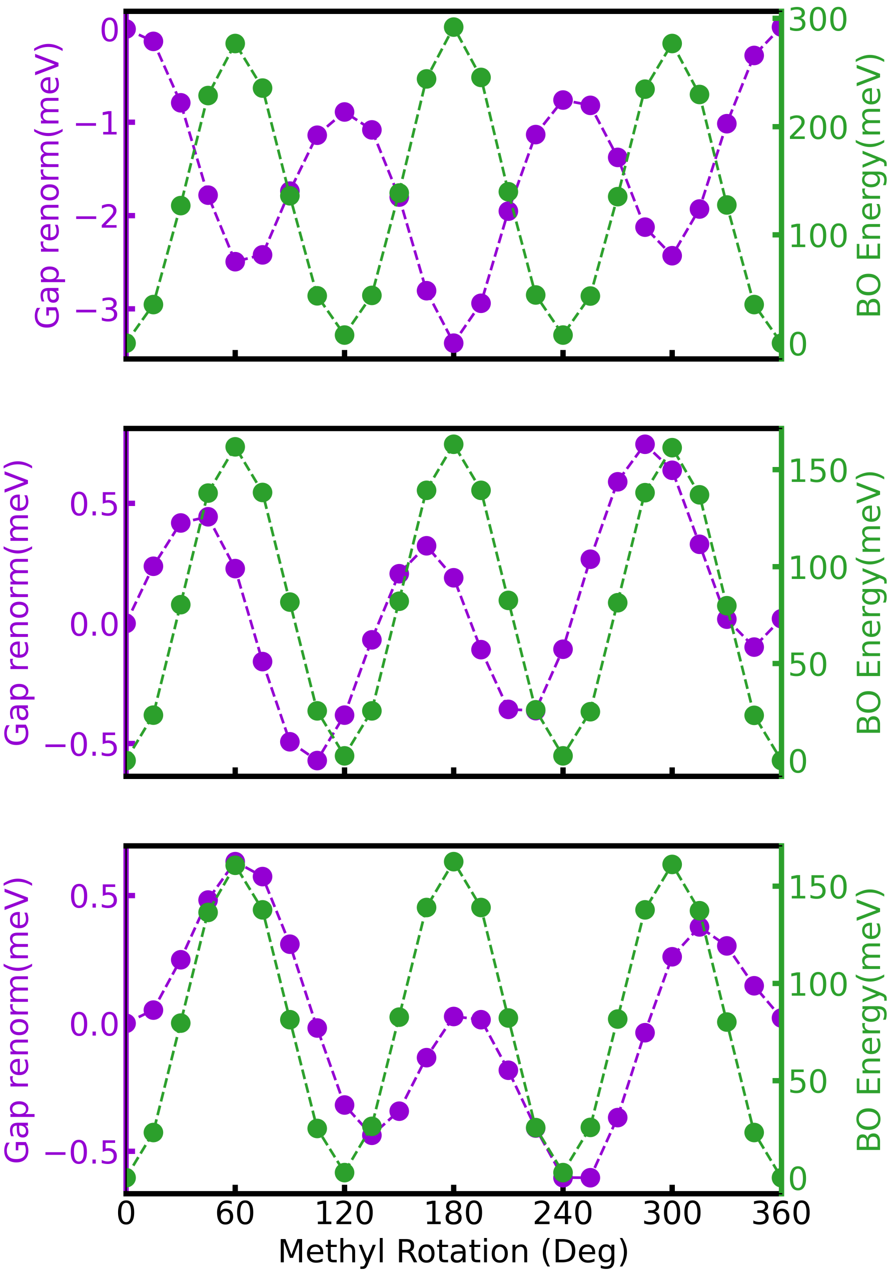

Figure S7: Born-Oppenheimer(BO) energies and the HOMO-LUMO gap renormalizations obtained with the PBE functional for an isolated NAI-DMAC molecule when its three methyl groups within the NAI unit are rotated relative to the geometry optimized position. The top, middle, and bottom panels show the results for the NAI methyl groups labeled with numbers 1, 2, and 3, respectively, in Fig. S6.

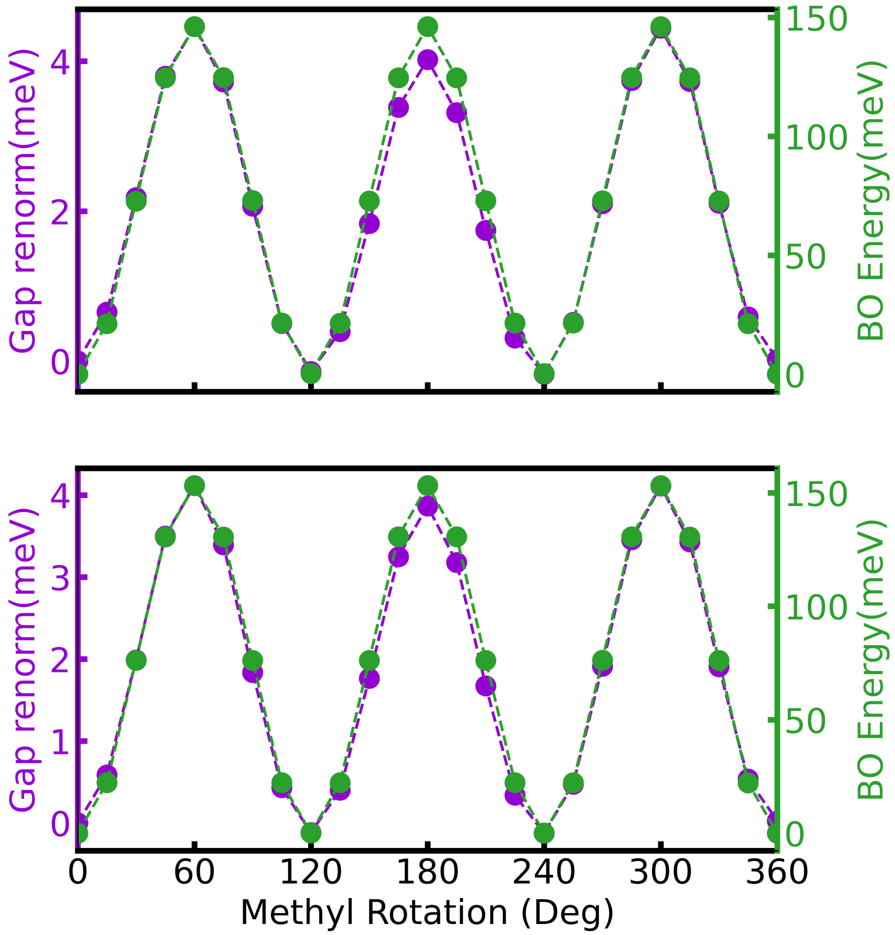

Figure S8: Born-Oppenheimer(BO) energies and the HOMO-LUMO gap renormalizations obtained with the PBE functional for an isolated NAI-DMAC molecule when its two methyl groups within the DMAC unit are rotated relative to the geometry optimized position. The top and bottom panels show the results for the DMAC methyl groups labeled with numbers 1 and 2, respectively, in Fig. S6.

References

(1) Monserrat, B. Electron–phonon coupling from finite differences. *J. Phys. Condens. Matter* **2018**, *30*, 083001.

(2) Berne, B. J.; Thirumalai, D. On the Simulation of Quantum Systems: Path Integral Methods. *Annu. Rev. Phys. Chem.* **1986**, *37*, 401–424.

(3) Herrero, C. P.; Ramírez, R. Path-integral simulation of solids. *J. Phys. Condens. Matter* **2014**, *26*, 233201.

(4) Ceriotti, M.; Bussi, G.; Parrinello, M. Nuclear Quantum Effects in Solids Using a Colored-Noise Thermostat. *Phys. Rev. Lett.* **2009**, *103*, 030603.

(5) Ceriotti, M.; Bussi, G.; Parrinello, M. Colored-Noise Thermostats à la Carte. *J. Chem. Theory Comput.* **2010**, *6*, 1170–1180.

(6) Huppert, S.; Plé, T.; Bonella, S.; Depondt, P.; Finocchi, F. Simulation of Nuclear Quantum Effects in Condensed Matter Systems via Quantum Baths. *Applied Sciences* **2022**, *12*.

(7) Monserrat, B. Vibrational averages along thermal lines. *Phys. Rev. B* **2016**, *93*, 014302.

(8) Zacharias, M.; Giustino, F. One-shot calculation of temperature-dependent optical spectra and phonon-induced band-gap renormalization. *Phys. Rev. B* **2016**, *94*, 075125.

(9) Han, P.; Bester, G. Large nuclear zero-point motion effect in semiconductor nanoclusters. *Phys. Rev. B* **2013**, *88*, 165311.

(10) García-Risueño, P.; Han, P.; Bester, G. Frozen-phonon method for state anticrossing situations and its application to zero-point motion effects in diamondoids. 2019.

(11) Francese, T.; Kundu, A.; Gygi, F.; Galli, G. Quantum simulations of thermally activated delayed fluorescence in an all-organic emitter. *Phys. Chem. Chem. Phys.* **2022**, *24*, 10101–10113.
